# Technology use and interest in digital apps for mental health promotion and lifestyle intervention among young adults with serious mental illness

**DOI:** 10.1016/j.jadr.2021.100227

**Published:** 2021-09-12

**Authors:** John A. Naslund, Kelly A. Aschbrenner

**Affiliations:** a Department of Global Health and Social Medicine, Harvard Medical School, 641 Huntington Avenue, Boston, MA, United States; b Department of Psychiatry, Geisel School of Medicine at Dartmouth, NH, Lebanon

**Keywords:** Young adults, Serious mental illness, Digital mental health, mHealth, Apps, Health promotion, Smartphone

## Abstract

**Background:**

Digital technology holds promise for reaching young adults with serious mental illness. This study seeks to characterize technology use and explore interests in digital health interventions among young adults with serious mental illness.

**Methods:**

A survey was collected from participants age 18–35 enrolled in a lifestyle intervention trial about their technology ownership and use; technology use for mental health or other health reasons; and interest in health apps.

**Results:**

Responses from 150 participants were summarized. Differences in technology use were compared between individuals with psychotic (*n* = 65) and non-psychotic disorders (*n* = 85). Most participants owned mobile phones (92%) and used social media (95%). Smartphone ownership was higher among participants with non-psychotic (98%) compared to psychotic (84%) disorders. Many participants searched online for information about their mental health (73%) or general health (79%). More participants with non-psychotic compared to psychotic disorders expressed interest in apps for depression (71% vs. 54%) or anxiety (78% vs. 54%). Interest in apps for lifestyle, behavioral health, and other health needs was similar between diagnostic groups.

**Limitations:**

These findings may not generalize to all young adults with serious mental illness.

**Conclusions:**

There is high access, use, and interest in technology among young adults with serious mental illness. This highlights potential for integrated digital interventions for mental and physical health in this high-risk group.

## 1. Introduction

Individuals living with serious mental illness face an early mortality disparity reflected by upwards of 30 years shorter life expectancy when compared to the general population (Liu et al., 2017). Mounting evidence supports the effectiveness of lifestyle interventions and health promotion programming that can successfully address risk factors such as cardiorespiratory fitness, sedentary lifestyle, dietary behaviors, and low mood and symptoms that get in the way of positive health behavior change (Firth et al., 2019). However, few individuals living with serious mental illness have access to these programs as part of routine mental health service delivery. Additionally, there has been less attention directed at addressing these risk factors in early adulthood, which could have greater impact in mitigating harmful consequences over the life course. Digital technologies hold promise for bridging these gaps, and could be used to extend the reach of mental health promotion and lifestyle interventions tailored to at-risk young adults with serious mental illness (Naslund and Aschbrenner, 2019).

The increasing access to and use of digital technologies including smartphones, online programs, and social media among individuals living with serious mental illness is well documented, as reflected by studies conducted across different community-based and clinical settings (Aschbrenner et al., 2018; Brunette et al., 2019; Firth et al., 2015; Naslund et al., 2016). In particular, research shows that young people with serious mental illness use popular technologies such as social media at comparable rates as the general population (Birnbaum et al., 2017), and express interest in accessing mental health services through these popular digital platforms (Beard et al., 2019; Naslund et al., 2019). This growing recognition of the use and interest in using technology among people with serious mental illness parallels the emergence of studies over the last decade demonstrating the feasibility, acceptability, and clinical benefits of digital technologies for individuals living with serious mental illness (Berry et al., 2016; Naslund et al., 2015). This includes smartphone-delivered interventions for improving symptom management, functioning and engagement in clinical care (Ben-Zeev et al., 2018), and monitoring symptom changes over time (Niendam et al., 2018).

More recently, there has been growing emphasis on the use of digital interventions specifically for reaching young adults with serious mental illness (Melbye et al., 2020). There have been preliminary feasibility and pilot studies of smartphone interventions for symptom tracking and improving functional outcomes (Bucci et al., 2018; Schlosser et al., 2018), as well as targeting loneliness and social anxiety (Lim et al., 2020; McEnery et al., 2021). This has also involved carefully tailoring the design and interface of digital interventions to appeal to the interests of younger demographic groups by incorporating peer moderators or interactive features that are similar to the look and feel of popular social media platforms (McEnery et al., 2021; Schlosser et al., 2016). Despite the early promise and acceptability of these digital interventions, as reflected by reports of high satisfaction among participants across many studies (Melbye et al., 2020), there remain several notable challenges. Many of the studies enrolling young adults with serious mental illness have included small feasibility or proof-of-concept studies, show difficulty in sustaining participant engagement over time, and have not been widely tested or implemented in routine service delivery settings (Melbye et al., 2020). Furthermore, there is ongoing need to sufficiently account for the characteristics and interests of the target group of participants in the design and delivery of these interventions (Mohr et al., 2017), as well as exploring opportunities for integrating strategies and content aimed at promoting both mental and physical health given that these are co-occurring health concerns among young adults with serious mental illness, and together contribute to significant reductions in life expectancy compared to the general population (Naslund and Aschbrenner, 2019).

To address these gaps in the literature, and to continue to advance the reach and impact of digital interventions for young adults with serious mental illness, it is necessary to better understand patterns of technology access and use in this high-risk group, as well as to capture insights about their interests in using technology for mental and physical health promotion programs. Successful digital interventions are frequently tailored to the needs of the target user group (Yardley et al., 2015), and for persons with serious mental illness this often means adaptations to the digital content and interface in order to accommodate users’ psychosocial context, cognitive functioning, and literacy levels (Ben-Zeev et al., 2014; Biagianti et al., 2017). It is also important to consider whether there may be differences in technology interest and use between diagnostic groups, recognizing that young adults with psychotic disorders such as schizophrenia spectrum disorders may have different priorities for using a mobile app and interests when compared to young adults with non-psychotic disorders such as mood disorders, anxiety disorders, or post-traumatic stress disorder. Furthermore, digital mental health technologies are often developed for specific mental illness diagnoses, making it necessary to consider if there are differences in preferences or interest in digital interventions between diagnostic groups. Therefore, the purpose of this exploratory study was to characterize the patterns and type of technology use and interest in digital interventions for promoting mental health and physical health among young adults with serious mental illness receiving care in community mental health centers. Specifically, our goal was to explore differences in technology access, use of different types of technology for mental health and physical health, and interest in digital interventions between participants with psychotic and non-psychotic disorders.

## 2. Methods

### 2.1. Participants and setting

Participants in this study were young adults (ages 18–35) with serious mental illness enrolled in the Fit Forward randomized clinical trial of a lifestyle intervention (ClinicalTrials.gov registration: NCT02815813). Details about the Fit Forward trial and methods are described elsewhere (Aschbrenner et al., 2018)(Aschbrenner et al., 2021). Participants were recruited from community mental health centers in the Northeastern United States from 2017 to 2019. Eligible participants were required to be receiving services, be at elevated cardiovascular risk defined as being overweight or obese based on body mass index (BMI) ≥25 kg/m^2^, and have a diagnosis of a serious mental illness defined in the DSM-5 as a psychotic disorder (i.e., schizophrenia spectrum disorders and psychotic disorders) or a non-psychotic disorder (i.e., mood disorders, anxiety disorders, or post-traumatic stress disorder). The current study reports on baseline data collected from participants on their use of technology. The Committee for the Protection of Human Subjects at Dartmouth College approved all study procedures.

### 2.2. Measures

Demographic and clinical characteristics were collected from participants at baseline. In addition, participants completed the Consumer Technology Use Survey, the primary measure of interest in this report. This measure was developed by our team based on prior research assessing technology use among individuals with SMI (Aschbrenner et al., 2018; Aschbrenner et al., 2019; Naslund et al., 2016) and covers three major topic areas related to technology use: (1) access and use of various types of digital technology including mobile phones, smartphones, the Internet and social media; (2) use of digital technology for health purposes, including for seeking information about mental health or physical health; and (3) interest in using smartphone apps for accessing programs for mental health, physical health, and other health reasons.

### 2.3. Statistical analysis

Participants’ responses to the Consumer Technology Use Survey were tabulated to provide summary statistics. Chi-square tests were used to explore differences in participants’ demographic characteristics and patterns of technology use, access, and interest in technology between participants with psychotic disorders and non-psychotic disorders. SPSS Software was used for all statistical analyses. *P*-values <0.05 were considered statistically significant.

## 3. Results

### 3.1. Demographic characteristics

Participant characteristics are listed in Table 1. Of 150 participants, about 43% had psychotic disorders (*N* = 65), and the remainder had non-psychotic disorders (*N* = 85), including mood disorders (37%), post-traumatic stress disorder (17%), and anxiety disorders (3%). The average age of participants was 28.38 years (SD=4.54), and 55% (*N* = 82) identified as being white and 26% (*N* = 39) as Black, with 30% (*N* = 45) identifying as being Hispanic. Greater proportions of participants with non-psychotic disorders were married (25% vs. 5%; *p* = 0.01), had completed some college or were college graduates (55% vs. 25%; *p* = 0.017), and were living independently (57% vs. 34%; *p* = 0.016) when compared to participants with psychotic disorders, respectively. Participants with psychotic disorders were more likely to be taking antipsychotic medication compared to participants with non-psychotic disorders.

### 3.2. Access and patterns of digital technology use

As outlined in Table 2, 92% of participants reported owning a mobile phone, with high rates of ownership observed in participants with psychotic (86%) as well as non-psychotic disorders (97%). Among participants who own mobile phones, most have smartphones (92%), though smartphone ownership was significantly higher among participants with non-psychotic compared to psychotic (98% vs. 84%; *p* = 0.007) disorders. Furthermore, frequency of mobile phone use and messaging differed between diagnostic groups. Participants with non-psychotic disorders were more likely to use their mobile phone daily (99% vs. 88%; *p* = 0.019), to send text messages several times each day (77% vs. 55%; *p* = 0.020) compared to participants with psychotic disorders. Participants with mood disorders were more likely to use their mobile phone to connect to the Internet (98% vs. 79%; *p* < 0.001) compared to participants with psychotic disorders, yet overall Internet use between diagnostic groups did not differ. Daily use of the Internet was higher among participants with non-psychotic disorders (96%) compared to participants with psychotic disorders (76%; *p* = 0.001). Most participants (95% of overall study sample) reported using social media. Interestingly, there were no statistically significant differences in use of social media between diagnostic groups, with most participants with non-psychotic (98%) and psychotic (92%) disorders using social media platforms. There were some differences in the types of platforms used between groups, though there were no significant differences in frequency of use between groups, with close to two thirds of participants reporting that they use social media on a daily basis.

### 3.3. Use of technology for mental health and other health concerns

Most participants reported having used the Internet to search for information about their mental health (73%) or general health (79%). As reflected in Table 3, a larger proportion of participants with non-psychotic disorders reported using the Internet to search for mental health information when compared to participants with psychotic disorders (78% vs. 66%, respectively), but this difference was not statistically significant. By contrast, a significantly greater proportion of participants with non-psychotic compared to psychotic disorders reported using the Internet to search for general health information (86% vs. 71%, respectively; *p* = 0.039). Though similar proportions of participants with either non-psychotic or psychotic disorders reported using social media platforms such as Facebook to search for information about their mental health (33% vs. 32%, respectively) and general health (40% vs. 35%, respectively).

There were few differences between diagnostic groups with regards to the types of digital health tools a doctor may have recommended for mental health or overall health, with one notable exception; nearly half of participants with non-psychotic disorders (48%) reported that their doctor recommended use of a crisis helpline when compared to about one third of participants with psychotic disorders (33%), a statistically significant difference (*p* = 0.044). Furthermore, a significantly larger proportion of participants with psychotic disorders (60%) reported that their doctor had never recommended any type of digital tools for mental health when compared to participants with non-psychotic disorders (40%; *p* = 0.021). With regards to doctor recommendations for digital tools for general health, there were no differences between diagnostic groups, though over 80% of participants indicated that their doctor had not recommended any digital technology for their health.

### 3.4. Interest in smartphone apps for mental health, lifestyle intervention, and other health reasons

**Fig. 1.**
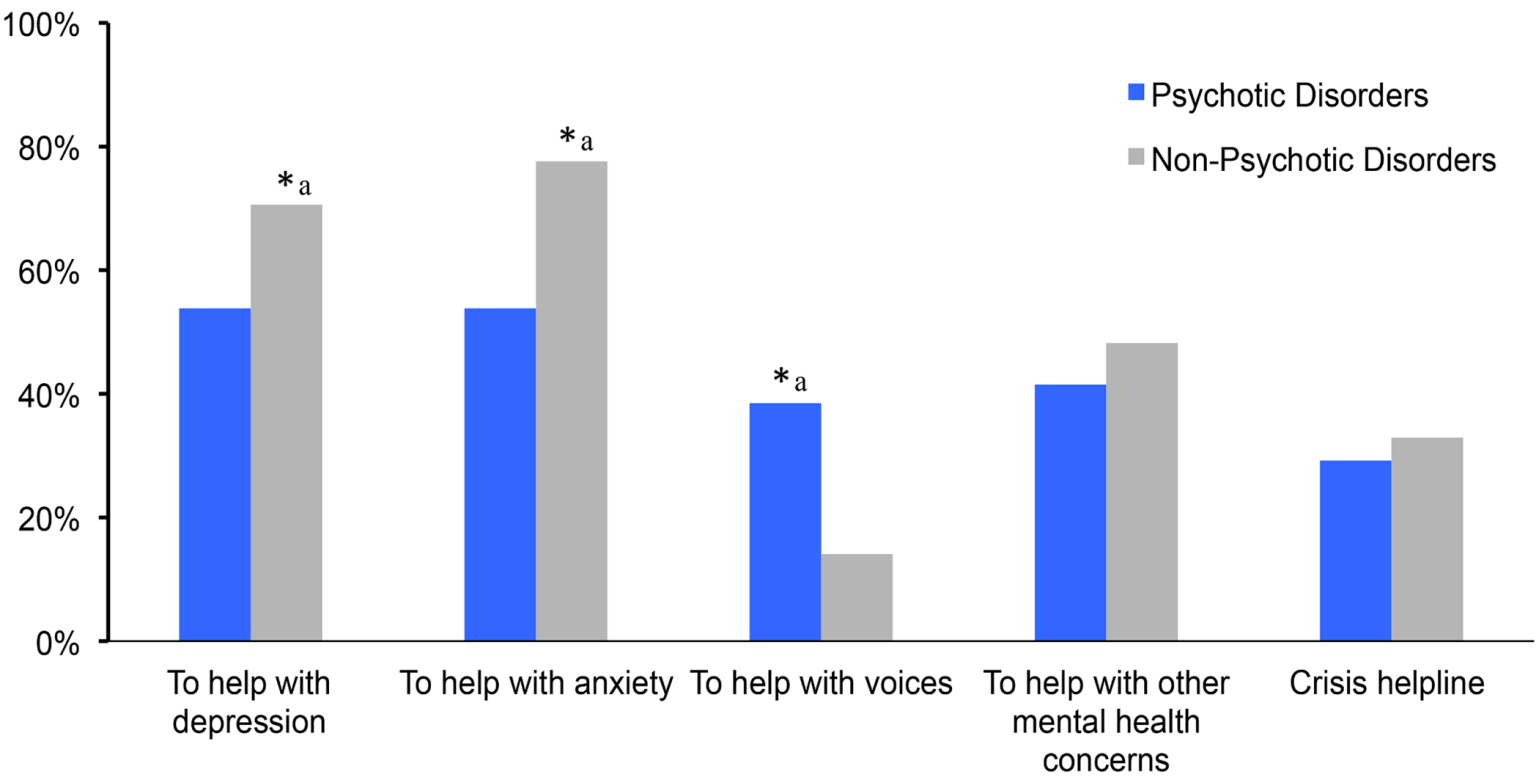
Participants’ interest in smartphone apps for mental health^a^ *p < 0.05 ^a^ Participants responded to the following question: “Would you be interested in any of these apps for mental health?”.

**Fig. 2.**
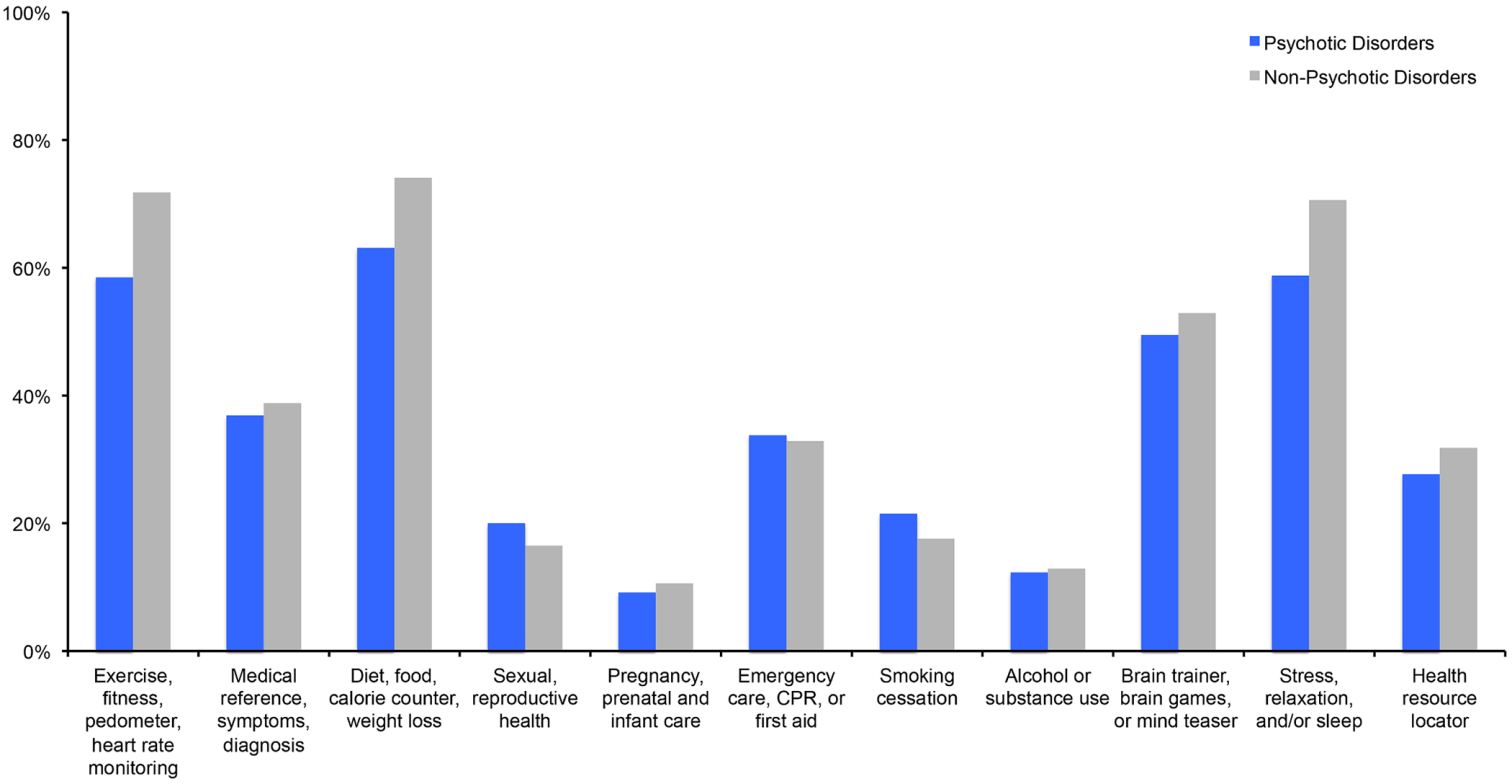
Participants’ interest in smartphone apps for health promotion and other health reasons^a^ *p < 0.05 ^a^ Participants responded to the following question: “Would you be interested in any of these health apps?”.

As illustrated in Fig. 1, in response to the following question: “*Would you be interested in any of these apps for mental health?*” a significantly greater proportion of participants with non-psychotic compared to psychotic disorders expressed interest in smartphone apps to help with depression (71% vs. 54%, respectively; *p* = 0.041) and anxiety (78% vs. 54%, respectively; *p* = 0.003). A significantly greater proportion of participants with psychotic disorders expressed interest in smartphone apps to help with voices when compared to participants with non-psychotic disorders (39% vs. 14%, respectively; *p* = 0.001). Whereas, as summarized in Fig. 2, in response to the following question “*Would you be interested in any of these health apps?*”, there were no significant differences in the selection of various health related apps between diagnostic groups. It is noteworthy that roughly two thirds of all participants’ expressed interest in apps for exercise or fitness (66%) or for diet (69%). Interestingly, over half of participants expressed interest in apps for cognition such as brain trainer or mind teaser games (51%), and apps for stress, relaxation, or sleep (65%).

## 4. Discussion

The findings described here are consistent with existing literature showing high access, use, and interest in mobile technology among young adults with serious mental illness (Brunette et al., 2019; Young et al., 2020), while also illustrating patterns of mobile technology use between individuals with psychotic and non-psychotic disorders. It is noteworthy that even though technology access was high across both diagnostic groups, significantly fewer individuals with psychotic disorders had access to smartphones, and their frequency of smartphone use and Internet use was lower when compared to individuals with non-psychotic disorders. Prior studies have found similar lower technology access in patients with psychotic disorders compared to non-psychotic disorders (Young et al., 2020), a gap that that may reflect various clinical or socio-demographic characteristics, such as more severe mental health symptoms, not living independently, or having lower level of educational attainment. For instance, the higher rates of independent living observed among participants with non-psychotic disorders may necessitate more frequent phone or Internet use, and thereby contribute to greater smartphone ownership in this diagnostic group. Interestingly, despite these differences, the proportion of participants who reported using social media, and the frequency of social media use did not appear to differ between diagnostic groups. This is also consistent with prior reports showing comparable patterns of social media use in young people with psychotic and non-psychotic disorders (Birnbaum et al., 2017).

In the current study, we also did not find any differences in access to and use of mobile technology between racial and ethnic minority groups. This is consistent with recent national survey data showing that Black and Hispanic young adults have comparable rates of smartphone ownership as whites (Pew Research Center, 2021b), as well as comparable or higher use of social media platforms as whites depending on the platform type (Pew Research Center, 2021c). These findings likely reflect the young age of our study sample, which is aligned with research showing that younger individuals with less education, lower-income, and from racial/ethnic minority groups are highly likely to be dependent on their smartphones (Tsetsi and Rains, 2017). It is also noteworthy that while we did not observe any differences between race and ethnic groups, that recent studies continue to illustrate that the digital divide persists in the United States in terms of access to and use of health-related technologies among older adults, whereby Blacks and Hispanics use technologies for health-related purposes less than whites (Mitchell et al., 2019). Furthermore, our study was focused on the use of mobile technologies, which could hold potential to bridge gaps in access and quality to mental health services (Friis-Healy et al., 2021), yet we did not capture details about use of home broadband internet, where there remain considerable gaps in access among low-income individuals, young people, and underrepresented racial and ethnic minority groups (Pew Research Center, 2021a; Singh et al., 2020). We also did not explore participants’ digital literacy, reflected as their skills or competencies required to effectively use and benefit from digital mental health tools, which is an emerging ‘second digital divide’ among individuals living with serious mental illness that likely parallels existing inequities in the use of digital technologies due to race and ethnicity, income, age, and education (Hoffman et al., 2020).

In this study, a large proportion of young adult participants with psychotic and non-psychotic disorders reported having searched for information about their mental health or physical health online. Roughly one third of participants across diagnostic groups also reported using social media to search for mental health or physical health information. This is consistent with high rates of searching for health information online and on popular social media among young adults from the general population (Fox and Duggan, 2013; Zhao and Zhang, 2017). Research from the general population has also demonstrated that individuals from lower income groups have significantly lower health literacy, and as a result, are less likely to search for health information online (Estacio et al., 2019). While we did not assess health literacy among participants in this study, our sample represents an at-risk patient group with serious mental illness, which is further reflected by the low rates of employment and low educational attainment. Our results suggest that even with potentially lower levels of health literacy, these young adult participants are actively using online technologies to seek information about their health. This is important to recognize because searching for health information online can be especially empowering for making sense of one’s own diagnosis and symptoms, and can help promote informed choices in seeking health care (Ziebland et al., 2004), while affording the flexibility to navigate between professional websites or medical resources, as well as more informal user-generated content and first person illness accounts posted on social media (Fergie et al., 2016). Yet, concerns have also been raised about the varying quality of online health information (Daraz et al., 2019), and specifically in studies with young adults, there have been challenges pertaining to the trust-worthiness and relevance of online mental health information (Gowen, 2013; Lai et al., 2018). Therefore, our study highlights an additional important need to consider what information these young adults may be finding online, how they are interpreting or using this information, and how best to support them in identifying reliable and trustworthy online resources for their mental and physical health needs.

When considering the role of clinicians, it is interesting that we found few differences between the diagnostic groups with regards to the types of digital health tools a doctor may have recommended for mental health or overall health. However, we found that a larger proportion of participants with non-psychotic disorders reported that their doctor had recommended use of a crisis helpline when compared to participants with psychotic disorders. This may reflect the greater lifetime risk of suicidal ideation that has been reported among individuals with mood disorders compared to psychotic disorders (Harvey et al., 2018). This difference could also reflect the wider availability of crisis helplines and well-established reputation of many of these services. Importantly, a larger proportion of participants with psychotic disorders relative to non-psychotic disorders indicated that their doctor had not recommended any type of digital tools for mental health. This may be reflective of the limited availability of quality mobile apps and other digital tools specifically designed for psychotic disorders. This is consistent with a recent review of commercial app marketplaces that found out of over 700 apps containing the terms “schizophrenia” or “psychosis” in the description, only 6 were clinical relevant and had supporting evidence (Lagan et al., 2020b). The remainder of the apps were mostly games, and some were even stigmatizing or derogatory towards persons living with psychotic disorders (Lagan et al., 2020b). Most participants across both diagnostic groups also indicated that their doctor had not recommended any digital tools for their general health. This appears to parallel the common disconnect in services for young adults with serious mental illness, where mental health care is typically separated from physical health care (Lawrence and Kisely, 2010). Digital health interventions could yield new opportunities to integrate evidence-based content aimed at simultaneously addressing physical and mental health needs of this high-risk patient group (Naslund and Aschbrenner, 2019).

Importantly, we found high interest in smartphone apps for mental health across both diagnostic groups. Over half of participants expressed interest in smartphone apps to help with depression and anxiety, with interest being substantially greater among participants with non-psychotic disorders. As expected, interest in smartphone apps for helping with voices was higher among participants with psychotic disorders. There was also comparable, and high interest in using smartphone apps for exercise, fitness or diet among participants. This is mostly likely reflective of participants’ enrollment in a clinical trial of a lifestyle intervention, where as a result, they were already motivated and interested in health promotion activities. Nevertheless, this is an important finding showing that young adult participants expressed interest in digital interventions for both their mental health and physical health. To date there has been less emphasis on using digital technology to address the physical health needs of young adults with serious mental illness. Recent studies have demonstrated the acceptability and initial effectiveness of digital programs for smoking cessation but that have mainly enrolled middle-age samples (Brunette et al., 2020; Vilardaga et al., 2020), while challenges have emerged with engaging persons with serious mental illness in online weight loss programs (Olmos-Ochoa et al., 2019). However, digital technology for health promotion among young adults with serious mental illness is an area ripe for further exploration. In particular, digital interventions could have a combined focus on physical health while accounting for mental health symptoms that create challenges for sustaining engagement and achieving benefits from these programs.

Given the detrimental effects of serious mental illness on cognitive function, as well as the frequently disrupted sleep patterns in this patient group, it is not surprising that we also found that there was high interest in apps for cognition, stress, and sleep across both diagnostic groups. Similarly, among young adults in the general population, a survey found that there was high interest in brain training apps (Torous et al., 2016); therefore, this could be a similar trend reflected among our young adult participants. Overall, these findings emphasize the potential to expand digital mental health apps to accommodate additional important health targets, which is a research area that is gaining momentum especially as wearable devices and sensors become more widely available, thereby enabling more reliable and continuous tracking of behaviors, cognition, mood, and sleep patterns (Aledavood et al., 2019; Torous et al., 2019).

Our findings of high use of technology for health reasons, combined with interest in using a wide range of health related apps for both mental health and physical health, suggest that young adults with serious mental illness are likely already searching commercial app marketplaces, either the Google Play store or iOS store, to find apps to meet their health needs. This raises important considerations for the digital mental health field, because despite promising scientific advances supporting the potential clinical benefits of digital mental health apps, the reporting of research findings substantially lags behind the commercial sector. There continues to be unprecedented growth in the number of available commercial digital mental health technologies, with well over 10,000 mental health apps available for download via commercial app stores (Torous et al., 2019). It is worrisome that so few of these commercially available apps are supported with reliable or high-quality scientific evidence, and in the most egregious examples even report false or misleading claims of clinical effectiveness (Larsen et al., 2019; Lau et al., 2020). To address this considerable challenge, there have been numerous calls for more robust standards to guide the evaluation and dissemination of mental health apps (Torous et al., 2019), as well as the recent dissemination of objective approaches for evaluating the quality of different features of mobile apps including safety, privacy, and accessibility (Lagan et al., 2020a).

### 4.1. Limitations

Several limitations with this study should be considered. Firstly, this was an exploratory study, and therefore was not sufficiently powered to test any specific hypotheses related to technology use and interest in technology in this patient group. Second, this study offers cross sectional findings, and as a result it is not possible to interpret participants’ interests in technology beyond what is reflected in their responses. Additional follow up interviews and focus group discussions could help to expand on the findings described here. For instance, with most participants’ in this study reporting generally high technology use, further in-depth exploration into how and when participants use their mobile devices, and whether there are socio-demographic differences, such as between gender or racial/ethnic minority groups, could offer valuable insights for informing the development and implementation of digital mental health interventions. Third, participants in this study were enrolled in a trial of a lifestyle intervention using digital technology, and therefore may have already been interested in using technology for either their mental health or physical health. Additionally, participants had elevated cardiovascular risk and were interested in health promotion, which may limit generalizability to other samples of young persons with serious mental illness. However, participants were not required to own or use technology at the time of enrolling in this study, with the only requirement that they be actively receiving services at either of study sites. While we recognize that this may be a limit to generalizability, this sample is reflective of young adult populations at risk of early mortality receiving services in community mental health settings.

## 5. Conclusion

This study adds to mounting evidence confirming the widespread access, use, and interest in digital technologies among individuals with serious mental illness. A novel contribution from this study is the emphasis on young adults with serious mental illness, a patient group that is often difficult to reach and engage in clinical services as well as research, and where digital technologies may be especially impactful in targeting the combination of mental health and behavioral health risk factors that contribute to early mortality earlier in the lifecourse. While this study also highlights notable differences in the patterns of technology use between young adults with psychotic compared to non-psychotic disorders, overwhelmingly there was consistent interest in digital interventions for both mental health and physical health across both groups. This highlights the potential for future studies to consider ways to support the integration of health promotion and lifestyle intervention content into existing digital mental health interventions, or vice versa, to meet the specific interests and health needs of this target user group. These findings also suggest that continued efforts are needed to support young adults with serious mental illness in accessing digital technologies, and in particular mobile apps, that are safe, reliable, and draw from an established scientific evidence-base, while considering whether young adults have the digital literacy and skills to fully benefit from the potential of these devices. This will require involvement of clinicians who are positioned to recommend apps to their clients, as well as additional resources to assist young people with serious mental illness in confidently navigating the vast commercial app marketplaces.

## Figures and Tables

**Table 1:** Baseline demographic characteristics of participants with psychotic disorders compared to non-psychotic disorders.

	Total Sample(*N* = 150)		Psychotic disorders(*N* = 65)		Non-Psychotic Disorders(*N* = 85)		*P*-value^a^
Characteristic	*N*	%	*N*	%	*N*	%	
Age (M ± SD)	28.38 ± 4.54		28.37 ± 4.40		28.39 ± 4.67		.980
Weight (M ± SD lbs)	106.38 ± 24.12		105.95 ± 19.06		106.71 ± 27.48		.843
Body mass index (M ± SD)	37.14 ± 7.4		36.88 ± 6.86		37.34 ± 8.55		.724
**Smoking status**							.736
Not Smoking	92	61.3	39	60	53	63.1	
Smoking	57	38.7	26	40	31	36.9	
**Birth sex**							.096
Male	64	42.7	33	50.8	31	36.5	
Female	86	57.3	32	49.2	54	63.5	
**Race**							.051
White	82	54.7	26	47.3	55	69.6	
Black	39	26	21	38.2	17	21.5	
Asian	1	7	1	1.8	0	0	
More Than One	12	8	7	12.7	7	8.9	
**Latino**							.472
Yes	45	30	22	33.8	23	27.4	
No	104	69.3	43	62.2	61	72.6	
**Marital Status**							**.01**
Married	13	8.7	3	4.6	21	25	
Never married	125	83.3	62	95.4	63	75	
**Education**							**.017**
Less than high school	22	14.7	12	18.7	10	11.8	
High school graduate	63	42	37	56.9	26	30.6	
Some college	50	33.3	14	21.5	36	42.4	
College graduate	15	10	2	3	11	13	
**Employment status**							.405
Working	28	18.7	10	15.4	18	21.2	
Not working	120	80	55	84.6	67	78.8	
**Residential**							**.016**
Living independently	70	46.7	22	33.8	48	56.5	
Living with family	43	28.7	23	35.4	20	23.5	
Supervised or supported housing	37	24.7	20	30.7	14	16.5	
**Contact with parents by Telephone**							.496
Not at all	13	8.7	6	9.2	7	8.3	
Everyday	64	4.3	28	43.1	36	42.9	
Once a week	40	26.8	21	32.3	19	22.6	
Once a month	18	12.1	6	9.2	12	14.3	
Less than once a month	14	9.4	4	6.2	10	11.9	
**Contact with parents by text message**							.117
Not at all	42	28.2	24	36.9	18	21.4	
Everyday	47	31.5	19	29.2	28	33.3	
Once a week	31	20.8	9	13.8	22	26.3	
Once a month	10	6.7	3	4.6	7	8.3	
Less than once a month	19	12.8	10	15.4	9	10.7	
**Contact with parents in person**							.129
Not at all	17	11.4	5	7.7	12	14.3	
Everyday	59	39.6	33	50.8	26	31	
Once a week	31	20.8	11	16.9	20	23.8	
Once a month	20	13.4	9	13.8	11	13.1	
Less than once a month	22	14.8	7	10.8	15	17.9	
**Taking antipsychotic medication**	106	70.7	59	92.2	47	56	<**.001**

a Bold face denotes statistical significance, defined as *P* value ≤0.05.

**Table 2:** Technology use among participants with psychotic disorders compared to non-psychotic disorders.

	Total Sample(*N* = 150)		Psychotic disorders (*N* = 65)		Non-Psychotic Disorders(*N* = 85)		*P*-value^a^
Mobile Phone Use	*N*	%	*N*	%	*N*	%	
**Do you have a mobile phone?**							.30
Yes	137	91.9	55	85.9	82	96.5	
No	12	8.1	9	14.1	3	3.5	
**What type of phone plan do you have?**							.533
Prepaid phone plan	40	29	9	33.9	21	25.6	
Post paid phone plan	9	6.5	3	5.4	6	7.3	
Monthly phone plan at fixed price	44	31.9	14	25	30	36.6	
Corporate phone plan	1	.7	1	1.8	0	0	
Family phone plan	38	27.5	16	28.6	22	26.8	
Don’t know/not sure	6	4.3	3	5.4	3	3.7	
**Is your mobile phone a smartphone?**							**.007**
Yes	127	92	47	83.9	80	97.6	
No	11	8	9	16.1	2	2.4	
**How often do you use your mobile phone?**							**.019**
Every day	130	94.2	49	87.5	81	98.8	
Several times each week	6	4.3	5	8.9	1	1.2	
Less than once per week	2	1.4	2	3.6	0	0	
**How often do you use your mobile phone to send or receive text messages (SMS)?**							**.020**
Several times each day	94	68.1	31	55.4	63	76.8	
Several times each week	35	25.4	22	39.3	13	15.9	
Less than once per week	5	3.6	2	3.6	3	3.7	
Never, I don’t use text messaging	4	2.9	1	1.8	3	3.7	
**Who do you share your mobile phone with?**							.580
Partners or spouse	3	2.2	1	1.8	2	2.4	
Parents	2	1.4	1	1.8	1	1.2	
Children	4	2.9	0	0	4	4.9	
Brother or sister							
Friends	3	2.2	1	1.8	2	2.4	
I do not share my mobile phone with anyone else	125	90.6	53	94.6	72	87.8	
**Do you use your mobile phone to connect to the Internet?**							<**.001**
Yes	124	89.9	44	78.6	80	97.6	
No	14	10.1	12	21.4	2	2.4	
**What type of operating system do you have on your smartphone?**							.508
Android	83	65.4	31	66	52	65	
Apple iOS	42	33.1	15	31.9	27	33.8	
Windows	1	.8	0	0	1	1.3	
Don’t know/not sure	1	.8	1	2.1	0	0	
**Do you have a tablet?**							.068
Yes	48	34.8	14	25	34	41.5	
No	90	65.2	42	75	48	58.5	
**What type of operating system do you have on your tablet?**							.761
Android	22	45.8	7	50	15	44.1	
Apple iOS	17	35.4	4	28.6	13	38.2	
Windows	2	4.2	1	7.1	1	2.9	
Don’t know/not sure	2	4.2	0	0	2	5.9	
Other	5	10.4	2	14.3	3	8.8	
***Internet Use***							
**Do you use the Internet?**							.318
Yes	145	97.3	62	95.4	83	98.8	
No	4	2.7	3	4.6	1	1.2	
**How often do you use the Internet?**							**.001**
Every day	127	87.6	47	75.8	50	96.4	
Several times each week	13	9	10	16.1	3	3.6	
**How do you typically connect to the Internet**							
My computer	61	40.7	31	47.7	30	35.3	.135
Family member’s or friend’s computer	18	12	9	13.8	9	10.6	.616
Computer at library	25	16.7	14	21.5	11	12.9	.188
Computer at workplace	9	6	5	7.7	4	4.7	.502
Computer at community center	28	18.7	15	23.1	13	15.3	.291
Mobile phone	131	87.3	49	75.4	82	96.5	**<.001**
Table (such as iPad)	31	20.7	14	21.5	17	20	.841
Internet café	13	8.7	5	7.7	8	9.4	.777
Other	4	2.7	0	0	4	4.7	.133
**Where do you typically access the Internet?**							
At home	129	86	56	86.2	73	85.9	1.0
Library or school	37	24.7	17	26.2	20	23.5	.849
Public areas with free WiFi	67	44.7	28	43.1	39	45.9	.744
Cafes or restaurants with free WiFi	53	35.3	18	27.7	35	41.2	.120
***Social Media Use***							
**Do you use social media?**							.240
Yes	143	95.3	60	92.3	83	97.6	
No	7	4.7	5	7.7	2	2.4	
**Do you use any of the following popular social media?**							
Facebook	121	80.7	49	75.4	72	84.7	.210
Twitter	30	20	12	18.5	18	21.2	.837
Instagram	67	44.7	21	32.3	46	54.1	**.009**
Snapchat	50	33.3	16	24.6	34	40	.056
YouTube	126	84	54	83.1	72	84.7	.826
LinkedIn	21	14	7	10.8	14	16.5	.353
Tumblr	9	6	3	4.6	6	7.1	.732
Pinterest	38	25.3	10	15.4	28	32.9	**.015**
Periscope	1	.7	0	0	1	1.2	1.0
Google Plus	19	12.7	10	15.4	9	10.6	.460
Reddit	15	10	4	6.2	11	12.9	.272
Other	8	5.3	2	3.1	6	7.1	.467
No, I don’t use social media	7	4.7	5	7.7	2	2.4	.240
**How often do you use any type of social media?**							.339
Every day	92	64.8	37	61.7	55	67.1	
Several times each week	35	24.6	14	23.3	21	25.6	
Less than once per week	15	10.6	9	15	6	7.3	
**What devices do you use to access social media?**							
Mobile phone	126	84	47	72.3	79	92.9	**.001**
Tablet	27	18	11	16.9	16	18.8	.832
Computer	59	39.3	31	47.7	28	32.9	.091
Other	3	2	1	1.5	2	2.4	1.0

a Bold face denotes statistical significance, defined as *P* value ≤0.05.

**Table 3:** Technology use for mental health and other health reasons among participants with psychotic disorders compared to non-psychotic disorders.

	Total Sample(*N* = 150)		Psychotic disorders (*N* = 65)		Non-Psychotic Disorders (*N* = 85)		*P*-value^a^
	*N*	%	*N*	%	*N*	%	
**Have you ever used the Internet to search for any information about your mental health?**							.130
Yes	106	73.1	41	66.1	65	78.3	
No	39	26.9	21	33.9	18	21.7	
**Have you ever used the Internet to search for any information about your own health?**							**.039**
Yes	115	79.3	44	71	71	85.5	
No	30	20.7	18	29	12	14.5	
**Have you ever used social media (e.g., Facebook) to search for information about your mental health?**							1.0
Yes	46	32.2	19	31.7	27	32.5	
No	97	67.8	41	68.3	56	67.5	
**Have you ever used social media (e.g., Facebook) to search for information about your health?**							.603
Yes	54	37.8	21	35	33	39.8	
No	89	62.2	39	65	50	60.2	
**Has your doctor ever recommended any of the following for your mental health?**							
Internet website	20	13.3	9	13.8	11	12.9	1.0
Smartphone/tablet app	14	9.3	3	4.6	11	12.9	.096
Specialized software	2	1.3	1	1.5	1	1.5	1.0
Crisis helpline	61	40.7	20	32.8	41	48.2	**.044**
Other	1	.7	0	0	1	1.2	1.0
None of the above	73	48.7	39	60	34	40	**.021**
**Has your doctor ever recommended any of the following for your health?**							
Internet website	14	9.3	7	10.8	7	8.2	1.0
Smartphone/tablet app	16	10.7	6	9.2	10	11.8	.096
Specialized software	2	1.3	1	1.5	1	1.2	1.0
Other	1	.7	1	1.5	0	0	.433
None of the above	121	80.7	52	80	69	81.2	1.0

a Bold face denotes statistical significance, defined as *P* value ≤0.05.
